# Increasing copper alters cellular elemental composition (Mo and P) of marine diatom

**DOI:** 10.1002/ece3.2890

**Published:** 2017-04-04

**Authors:** Deli Wang, Weiwei Xia, K. Suresh Kumar, Kunshan Gao

**Affiliations:** ^1^ State Key Laboratory of Marine Environmental ScienceXiamen UniversityXiamenChina; ^2^ Department of BotanyUniversity of AllahabadAllahabad 211002India

**Keywords:** algae, copper, elemental interactions, molybdenum, phosphorus

## Abstract

The elemental composition (surface adsorbed and internalized fraction of Cu, Mo and P) in marine phytoplankton was first examined in cultures of the diatom *Phaeodactylum tricornutum* which were exposed to various levels of Cu concentrations ranging from 0.25 to 16 μmol/L with equivalent free [Cu^2+^] concentrations of 0.4–26 nmol/L. We observed an acceleration of algal growth rates (20–40%) with increasing ambient Cu levels, as well as slightly increased levels of internalized Cu in cells (2–13 × 10^−18^ mol/cell) although cellular Cu mostly accumulated onto the cell surface (>50% of the total: intracellular + surface adsorbed). In particular, we documented for the first time that the elemental composition (Mo and P) in algal cells varies dynamically in response to increased Cu levels: (1) Cellular P, predominantly in the intracellular compartment (>95%), shows with a net consumption as indicated by a gradual decrease with increasing [Cu^2+^] (120→50 × 10^−15^ mol P/cell) probably due to the fact that P, a backbone bioelement, is largely required in forming biological compartments such as cell membranes; and (2) cellular Mo, predominantly encountered in the intracellular compartment, showed up to tenfold increase in concentration in the cultures exposed to Cu, with a peak accumulation of 1.1 × 10^−18^ mol Mo/cell occurring in the culture exposed to [Cu^2+^] at 3.7 nmol/L. Such a net cellular Mo accumulation suggests that Mo might be specifically required in biological processes, probably playing a counteracting role against Cu.

## 1 Introduction

Copper (Cu) essentially forms enzyme cofactors in the photosynthesis, nitrogen fixation, and other processes which result in marine phytoplankton growth, while dissolved Cu concentrations exceeding tolerance levels have a toxic effect on phytoplankton (Morel & Price, 2003; Sunda, 1975; Sunda, 2012). Generally, the toxicity and bioavailability of the metal are usually species specific, not depending on the total dissolved pool, but rather on the free metal ions (e.g., Leao, Vasconcelos, & Vasconcelos, 2007; Sunda & Lewis, 1978). Suitable amount of ambient Cu^2+^ ions generally triggers the growth of phytoplankton in seawater. On the other hand, high levels of Cu^2+^ might also hinder the physiological activities of phytoplankton. For example, most cyanobacteria experience reduced reproduction rates at [Cu^2+^] levels of >10^−11^ mol/L, and eukaryotic algae at [Cu^2+^] concentrations of 10^−10^–10^−8^ mol/L (Bruland, Donat, & Hutchins, 1991; Kozelka & Bruland, 1998). Previous studies suggest that excess Cu^2+^ ions interfere the primary reactions of photosynthesis and inhibit photosystem II and photosynthetic electron transport (Cid, Herrero, & Torres, 1995; Jegerschöld et al., 1995; Nielsen, Brownlee, Coelho, & Brown, 2003; Schröder et al., 1994; Yruela, Pueyo, Alonso, & Picorel, 1996). In addition, high levels of Cu can interfere with the cellular uptake of other elements such as Fe (Arredondo, Martínez, Núñez, Ruz, & Olivares, 2006; Lidon & Henriques, 1993), P (Peterson, Healey, & Wagemann, 1984), and Mn and Zn (Sunda & Huntsman, 1988).

Phytoplankton species adopt cellular mechanisms, when facing high levels of metals, that lower the free metal levels, by excreting organic substances, complexing metals extracellularly (Croot, Moffett, & Brand, 2000; Gordon, Howell, & Harwood, 1994; Hall, 1981). Other phytoplankton species sequestrate Cu in polyphosphate granules (Ahner & Morel, 1995; Jensen, Baxter, Rachlin, & Jani, 1982; Raskin, Kumar, Dushenkov, & Salt, 1994), or bind and precipitate Cu within the cytoplasm and/or vacuole (Reed & Gadd, 1990). Some phytoplankton species even produce chaperon proteins detoxifying reactive oxygen species inside the cells (Mehta & Gaur, 1999). As a result, many phytoplankton species can generally grow well even under moderately high levels of metals in natural waters.

Over the past decades, dissolved metals (including Cu) are subject to increase in coastal waters probably due to anthropogenic discharges from bottom paints on vessels, sedimentary releases (Beck & Sañudo‐Wilhelmy, 2007), and ocean acidification (Hoffmann, Breitbarth, Boyd, & Hunter, 2012; Millero, 2009). Marine phytoplankton species are being subjected to increased stress resulting from facing an increasing pressure of metal availability or toxicity such as that from free Cu^2+^. However, the authors have not encountered any reports in the literature analyzing the effects of Cu concentration levels on elemental composition ratios, as well as accounting for subsequent molybdenum (Mo) and phosphorus (P) responses in marine phytoplankton. *Phaeodactylum tricornutum*, a fast‐growing diatom which exists widely in coastal waters and is also commonly used as aquaculture feed (Duerr, Molnar, & Sato, 1998), does not have an effective mechanism for eliminating Cu from its cells (Angel, Simpson, Chariton, Stauber, & Jolley, 2015). Hence, there is a possibility of negative impact on higher levels of the trophic food chain. Previous studies have found that such effects of Cu are concentration range dependent. At low levels, it functions as a nutrient, while at higher ones is toxic with incremental physiological activities (e.g., Bentley‐Mowat & Reid, 1977; Cid et al., 1995). *Phaeodactylum tricornutum* generally synthesizes phytochelatins (glutathione and glutathione‐related peptides) sequestering metals including Cu intracellularly (e.g., Grill, Winnacker, & Zenk, 1985; Rijstenbil & Wijnholds, 1996). *Phaeodactylum tricornutum* may also excrete thiolic compounds complexing Cu extracellularly (Vasconcelos & Leal, 2008).

Our study initially examined the growth rates, physiological parameters, and intracellular and extracellular elements (Cu, P and Mo) in cultures of the marine diatom *P. tricornutum* while increasing Cu concentrations, with the objective of exploring phytoplankton cellular response that affected its elemental composition, as well as subsequent, resultant, and derivative ecological consequences due to increased pressure of dissolved metals (Cu^2+^) in coastal waters.

## 2 Methods

### 2.1 Cell culture and experimental design

*Phaeodactylum tricornutum* was obtained from the Center for Collections of Marine Bacteria and Phytoplankton of the State Key Laboratory of Marine Environmental Sciences (Xiamen University). The cells were acclimated to each Cu level for more than 10 generations before being used. Culturing was carried out in filtered seawater (0.2‐μm polycarbonate filters) taken from the South China Sea (116 E, 18 N) with Aquil medium enrichment. Culture solutions for cultures were carefully boiled to eliminate any possibility of bacterial contamination (e.g., Gao et al., 2012; Li, Gao, Villafañe, & Helbling, 2012; Li, Xu, & Gao, 2014). The background level of dissolved Cu in the seawater was verified to have a concentration of <1 nmol/L using the method of Wang et al. (2012). The additions of dissolved Cu to each culture were 0, 0.25, 0.5, 1, 2, 4, 8, and 16 μmol/L. The total dissolved Cu was maintained at relatively constant concentrations throughout the experiments.

Cu toxicity is impressed on marine phytoplankton mainly through the effects of free cupric ions instead of the total dissolved Cu (Bruland et al., 1991; Whitfield, 2001). In our study, we estimated the concentrations of free cupric ions (Cu^2+^) in the culture media based on the total dissolved Cu and therefore obtained concentrations of free cupric ions in the culture media in the range of 0.5–25 nmol/L. Other parameters such as pH, salinity, and EDTA (MINEQL+, version 4.6) were also evaluated.

Cultures were maintained at 20 ± 1°C in an incubator using cool fluorescent light at a level of 40 μmol/m^2^/s (12L:12D). Filtered air was constantly bubbled through the cultures, and the cells were harvested at 72 hr and then used for all measurements. A triplicate sample set was cultured for each concentration level of ambient Cu.

### 2.2 Quantum yield (Fv′/Fm′) and maximum relative electron transfer rate (rETR_max_) measurements

Fν′/Fm′ is the maximum quantum efficiency and reflects the probability of PSII reaction centers to use the available excitation energy for photochemistry. rETR_max_ is the maximum relative electron transfer rate of PSII photochemistry. The quantum yield of cells (Fv′/Fm′) and rETR_max_ of photosystem II in each culture were measured with an XE‐PAM (Walz, Germany) with the saturation light at 5,000 μmol photons m^−2^ s^−1^ for 0.8 s. Parameters of the rETR vs. the irradiance I (μmol m^−2^ s^−1^) curves were analyzed as: rETR = *I*/(*aI* ^2^ + *bI* + *c*), where a, b, and c are the adjustment parameters. The initial slope (i.e., α) of the maximum rETR (rETR_max_) was then expressed as a function of the parameters *a*,* b*, and *c*: rETR_max_ = 1/[*b* + 2(*ac*)^1/2^] (Gao et al., 2012).

### 2.3 Cell abundance and growth rates

Cell numbers were obtained using a Z2TM Coulter Counter (Beckman, Buckinghamshire, UK). The growth rates were determined based on the cell number changes and were calculated using the equation: μ = (ln*N*_1_−ln*N*_0_)/(*t*_1_–*t*_0_), where *N*_1_ and *N*_0_ are the cell concentrations at the culture time of *t*_1_ and *t*_0_. Growth rates were calculated based on cell abundance measurements of 11–12 counts for each culture.

### 2.4 Intracellular and extracellular elements (Cu, P, and Mo)

Two aliquots of water samples were taken at each addition of Cu during the experiments in order to study the kinetics of adsorption and uptake. One set of samples was filtered through 0.2‐μm polycarbonate filter membranes held in polypropylene filter sandwiches to determine the total cellular elements (Cu, P, and Mo) (the sum of intracellular and extracellular). The other set of samples was filtered identically, and the cells retained by the filters were washed with 5 ml of an oxalate solution to remove surface‐adsorbed metals (Tovar‐Sanchez et al., 2003) and rinsed with filtered, Chelex‐cleaned seawater (Tang & Morel, 2006). All samples were taken at the end of each culture. Materials on the total and intracellular filters were digested at room temperature with 2 ml ultrapure aqua regia and 50 μl HF. Concentrated acids were evaporated to dryness and reconstituted with 2 ml of 1N ultrapure HNO_3_. Total and intercellular metal extracts were analyzed using direct injection in an Agilent ICP‐MS (7700) at Xiamen University following tenfold dilution, with indium as an internal standard. Extracellular elements were then obtained by subtracting the intracellular result from the total.

## 3 Results and Discussion

The growth rates, physiological parameters, and cellular elements of *P. tricornutum* are summarized in Table 1. Generally, our results showed a healthy growth of *P. tricornutum* exposed to [Cu^2+^]. A three times increase in cell abundances and 40% in growth rates were observed in cultures exposed to Cu of 8–16 μmol/L. A slight inhibition on physiological activities was observed in cultures exposed to [Cu^2+^] as indicated by a slight decrease of Fv′/Fm′ and rETR_max_ (by 10% and 15%), although no inhibition on the growth. More importantly, cellular elemental composition was greatly altered especially in the early stage: Along with increased accumulation of Cu onto the cell surface (50–80%), intracellular P slightly decreased but intracellular Mo was significantly elevated by as much as tenfold in response to increased Cu levels.

**Table 1:** Cell abundances, growth rates, physiological states, and cellular elements (Cu, P, and Mo) (average ± *SD*,* n* = 3) in 72‐hr cultures of *Phaeodactylum tricornutum* at different levels of Cu stress

Dissolved Cu [μmol/L]	Free Cu^2+^ [nmol/L]	Abundances [10^5^ cells/ml]	Growth rate [per day]	Fv/Fm	rETR_max_	Total cellular Cu [10^−18^ mol/cell]	Surface‐adsorbed Cu [10^−18^ mol/cell]	Intracellular Cu [10^−18^ mol/cell]	Cellular P [10^−15^ mol/cell]	Cellular Mo [10^−18^ mol/cell]
0.00	0.0	3.2 ± 0.2	0.54 ± 0.01	0.55 ± 0.01	71 ± 1.1	4.2 ± 1.6	4.2	1.3 ± 1.0	122 ± 11	0.14 ± 0.09
0.25	0.4	3.1 ± 0.2	0.53 ± 0.01	0.55 ± 0.01	69 ± 3.6	5.6 ± 1.2	2.8	2.8 ± 2.4	95 ± 10	0.31 ± 0.13
0.50	0.8	5.9 ± 0.7	0.69 ± 0.02	0.52 ± 0.01	63 ± 1.8	9.9 ± 7.5	8.4	1.5 ± 0.5	53 ± 2	0.08 ± 0.03
1.0	1.8	6.0 ± 0.3	0.69 ± 0.01	0.51 ± 0.00	59 ± 1.0	14 ± 1.8	11.4	2.6 ± 0.2	54 ± 5	0.71 ± 0.21
2.0	3.7	5.6 ± 0.2	0.60 ± 0.01	0.49 ± 0.03	66 ± 1.3	25 ± 4.1	21	4.0 ± 2.4	86 ± 4	1.10 ± 0.33
4.0	7.6	5.1 ± 0.2	0.58 ± 0.01	0.51 ± 0.01	60 ± 3.6	32 ± 5.3	26.5	5.5 ± 1.0	93 ± 10	–
8.0	15	9.7 ± 2.6	0.74 ± 0.07	0.51 ± 0.00	60 ± 1.4	40 ± 4.4	32.4	7.6 ± 1.2	47 ± 0	0.62 ± 0.09
16	26	8.3 ± 1.2	0.70 ± 0.04	0.51 ± 0.00	60 ± 2.5	53 ± 11	40.2	12.8 ± 1.3	49 ± 2	0.19 ± 0.10

### 3.1 Sensitivity and tolerance of *Phaeodactylum tricornutum* to Cu

High levels of Cu exert inhibitory effects on marine phytoplankton, and these effects are generally species specific. A Cu concentration of 0.10 mg/L provokes approximately a 50% growth reduction and 1 mg/L inhibits the growth of *P. tricornutum* (Cid et al., 1995). Diatoms *Achnanthes brevipes* and *Cylindrotheca closterium* could tolerate dissolved Cu of as high as 0.5 mg/L without significant inhabitation on growth (Pistocchi, Mormile, Guerrini, Isani, & Boni, 2000). On the other hand, phytoplankton also develops defense mechanisms against toxic metals. The toxicity can be largely reduced extracellularly if the toxic Cu^2+^ions are complexed with organic compounds (Fisher & Frood, 1980) and exudates (Gnassia‐Barelli et al., 1978). High levels of EDTA in our culture media (Aquil) could have greatly complexed with free Cu^2+^, reducing the possibility of the metal binding with cell surface sites (Les & Walker, 1984), and thus the toxicity of Cu. After long‐term acclimation, Wikfors and Ukeles (1982) observed that coastal algae adapt to Cu levels of as high as 47.3 mg/L. Bentley‐Mowat and Reid (1977) reported that *P. tricornutum* can survive at Cu of as high as 10^−3^ mol/L.

**Figure 1:**
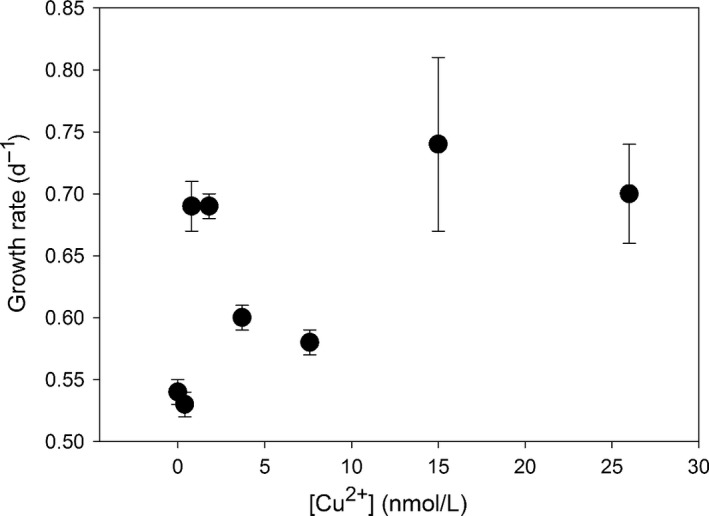
Growth rates of *Phaeodactylum tricornutum* in 72‐hr cultures exposed to [Cu^2+^]

We did not observe any significantly decreased growth of *P. tricornutum* (Figure 1), which suggests that the range of dissolved Cu in our study was far less than those toxic levels. Indeed, the initial cell abundance in each culture was ~0.8 × 10^5^ cells/ml. The final cell abundance reached ~3 × 10^5^ cells/ml in the culture without Cu exposure, a higher cell abundance of 5–6 × 10^5^ cells/ml at [Cu^2+^] levels of 0.4–7.6 nmol/L, and highest cell abundance of 8–9 × 10^5^ cells/ml at [Cu^2+^] levels of 15–26 nmol/L. Consistently, the growth rates of *P. tricornutum* also increased with increasing [Cu^2+^]: μ: ~0.5 per day in the control, but as high as 0.7 per day (an increase of 40%) in the culture exposed to Cu levels of 15 nmol/L (Figure 1). The results show that marine diatom such as *P. tricornutum* could grow well at [Cu^2+^] of as high as 26 nmol/L (Figure 1). Indeed, the diatom increased the Cu uptake and growth along with the increasing ambient Cu. Our results particularly point out that a slight increase of ambient [Cu^2+^] could trigger the growth of certain phytoplankton species such as *P. tricornutum*.

Cu is a constituent of the primary electron donor in photosystem I (PSI) and aids in the regulation of PSII‐mediated electron transport. On the other hand, higher levels of Cu ions can severely inhibit the photosynthetic electron transport rate in PSII (Barón, Arellano, & Gorgé, 1995; Cid et al., 1995; Gledhill, Nimmo, Hill, & Brown, 1997; Janik, Maksymiec, & Gruszecki, 2010), as the complex action of Cu^2+^ may primarily target the reaction center of PSII (Yruela et al., 1996). Severe environmental stresses, in the presence of light, commonly cause a significant decrease of Fν′/Fm′ and rETR_max_, and therefore, Fv′/Fm′ and rETR_max_ have been used as a sensitive indicator of photosynthetic stress (Baker, 2008; Li, Wai, Li, Coles, & Ramsey, 2000).

**Figure 2:**
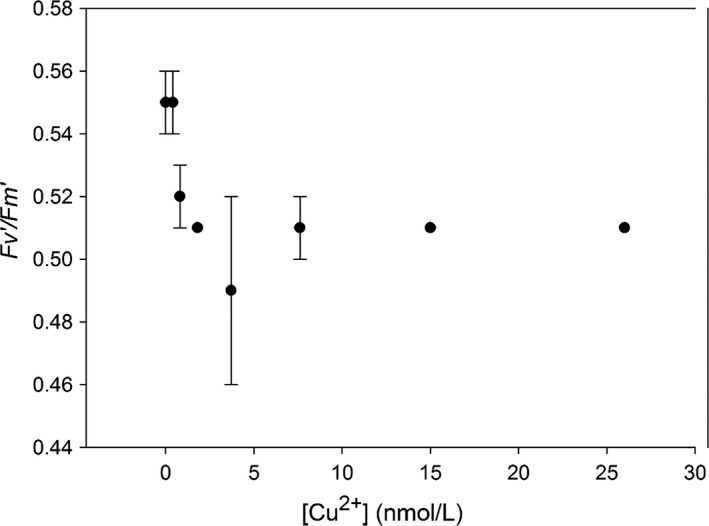
Fv′/Fm′ dynamics in the cultures of *Phaeodactylum tricornutum* exposed to increasing [Cu^2+^]

**Figure 3:**
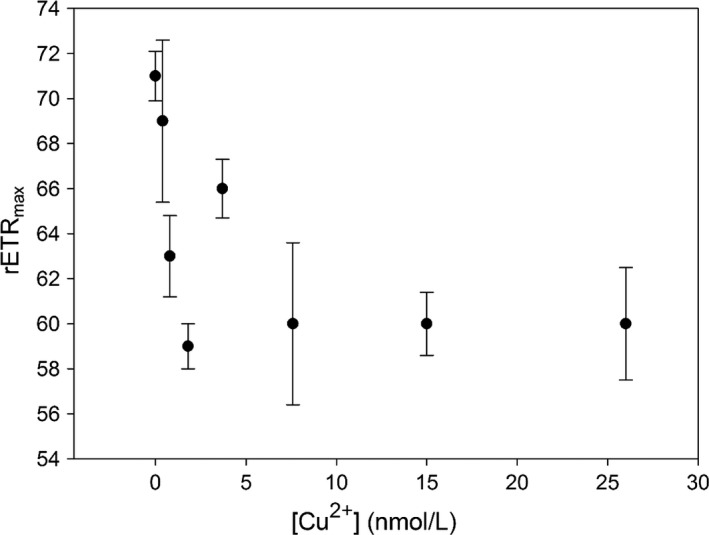
rETRmax dynamics in the cultures of *Phaeodactylum tricornutum* exposed to increasing [Cu^2+^]

Our cultures of *P. tricornutum* were characterized by relatively high *F*ν′/*F*m′ values (0.55–0.50) (Figure 2) and high rETR_max_ (0.70–0.50) (Figure 3), indicative of a relatively well functioning PSII in *P. tricornutum*. Our results further showed only a slight depression of Fv′/Fm′ (0.55–0.51) and rETR_max_ (0.70–0.60) of *P. tricornutum* in the culture exposed to [Cu^2+^] of 0.4 nmol/L. The depression increased slightly in cultures exposed to [Cu^2+^] of 3.7–26 nmol/L, as indicated by a decrease of the Fv′/Fm′ by 10%, and the rETR_max_ by 15%. The results reflected that PSII photochemistry was relatively sensitive to Cu stress, and a slight depression could occur even in cultures exposed to slightly increased [Cu^2+^] (e.g., 0.4 nmol/L), although with no hindrance to the growth rate.

### 3.2 Adsorption and uptake of Cu by phytoplankton

**Figure 4:**
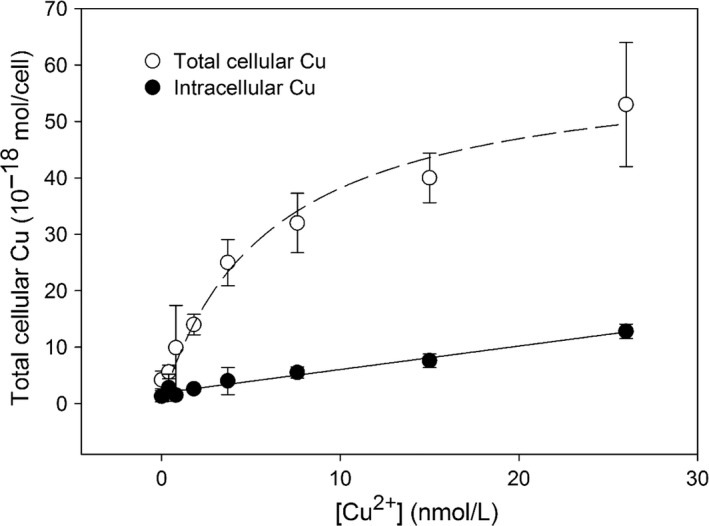
Kinetics of total cellular Cu and intracellular Cu by *Phaeodactylum tricornutum* in cultures exposed to [Cu^2+^]. The dashed line represents a Michaelis–Menten hyperbolic fitting of total cellular Cu with [Cu^2+^], and the solid line represents the linear fitting of intracellular Cu with [Cu^2+^]

Total cellular Cu could be divided into two compartments: surface adsorbed and intracellular. Our results showed that intracellular Cu ranged from 1.3–12.8 × 10^−18^ mol/cell, while total cellular Cu (intracellular + extracellular) ranged from 4.2–53 × 10^−18^ mol/cell (Figure 4). The intracellular Cu in *P. tricornutum* observed in our study was far less than that in phytoplankton showing Cu toxic symptoms, for example, *Dunaliella tertiolecta* (0.93 ± 0.08 × 10^−15^ mol Cu/cell), *P. tricornutum* (0.36 ± 0.30 × 10^−15^ mol Cu/cell), and *Tetraselmis sp*. (0.30 ± 0.01 × 10^−15^ mol Cu/cell) (exposed to 0.8 μmol Cu/L, Levy et al., 2008), and 10^−14^ to 10^−12^ mol Cu/cell in some marine algae (Debelius, Forja, DelValls, & Lubián, 2009). Instead, our results were comparable to those phytoplankton without Cu exposure, for example, 0.9–21 × 10^−18 ^mol/cell (Quigg et al., 2003), 0.2–18 × 10^−18 ^mol/cell (Hudson & Morel, 1993), and 3.8 × 10^−18 ^mol/cell (Sunda & Huntsman, 1995). Similar levels of cellular Cu as in these healthy cells confirm that *P. tricornutum* in our cultures maintained a relatively healthy range of intracellular Cu even when exposed to [Cu^2+^] of 0.4–26 nmol/L. Such results could be simply attributed to little toxic effects of Cu concentrations used in our study.

Here, our study shows that surface‐adsorbed Cu accounted for 50–80% of the total cellular Cu (Table 1; Figure 4). Cellular Cu was found to be adsorbed mostly on the cell surface (80%) in cultures exposed to low [Cu^2+^]: 0.4–3.7 nmol/L (Table 1). With further increasing [Cu^2+^] (3.7–26 nmol/L), more Cu ions were adsorbed to the cell surface and further subject to be transported into the cells, leading to a decrease of the surface‐adsorbed Cu (~50%) (Table 1). High surface adsorption of Cu might be attributed to a combination of adsorption onto algal surfaces, and possibly complexation with algal exudates (Levy, Stauber, & Jolley, 2007). We simply modeled the processes of Cu accumulation in phytoplankton as in two steps: adsorption on the cell surface and internalization into the cells. Our model shows that uptake of total cellular Cu from [Cu^2+^] in the media follows a Michaelis–Menten hyperbolic fitting:

$$C=C_{\max}\times \left[{\text{Cu}}^{2+}\right]/\left(K_{m}+\left[{\text{Cu}}^{2+}\right]\right)$$

where *C* is the total cellular Cu concentration, *C*_max_ is the maximum concentration of total cellular Cu, and *K*_m_ is the half‐saturation concentration. Therefore, we obtained the values for *C*_max_ and *K*_m_ by the least square fitting, as 61.0 × 10^−18^ mol/cell and 5.97 nmol/L (Figure 4). Internalization of Cu from the surface into the cells follows linear saturation kinetics, as consistent with previously reported models (Hudson & Morel, 1990; Knauer, Behra, & Sigg, 1997). The linear equation of intracellular Cu with [Cu^2+^] is as follows:

$$\begin{array}{cc} \text{Intracellular Cu}\;(10^{-18}\;\text{mol}/\text{cell})\;= & \;0.42\;\times \;\left[{\text{Cu}}^{2+}\right]\;\left(\text{nmol}/L\right)\;+\\ & \;1.9,\;r^{2}\;=\;.98,\;p\;<\;.01] \end{array}$$

The linear saturation kinetics between intracellular Cu and [Cu^2+^] suggests that [Cu^2+^] of 3.7–26 nmol/L is still far away from reaching the maximum concentration of transport ligands on the cell surfaces of *P. tricornutum*, as consistent with our previous analysis that *P. tricornutum* is subject to increased growth rate under increasing ambient Cu, instead of toxic effects.

### 3.3 Possible physiological involvements of P and Mo in *Phaeodactylum tricornutum*

**Figure 5:**
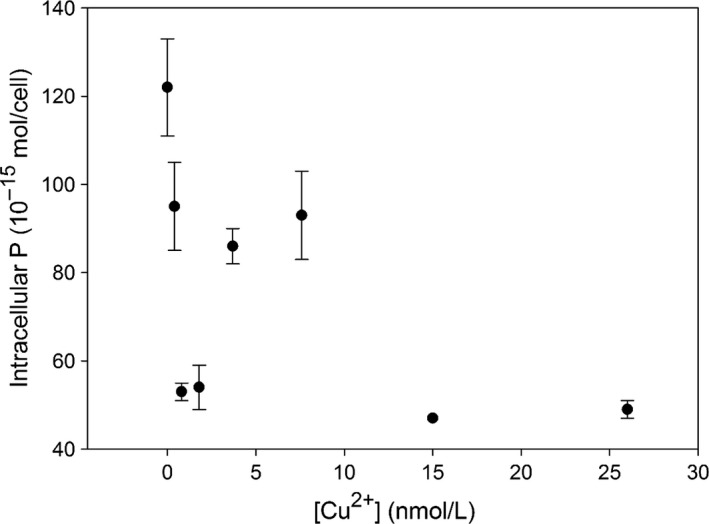
Intracellular P vs. free Cu^2+^ in the culture media. The dashed and solid lines reflect the effects of intracellular Cu on intracellular P and Mo

**Figure 6:**
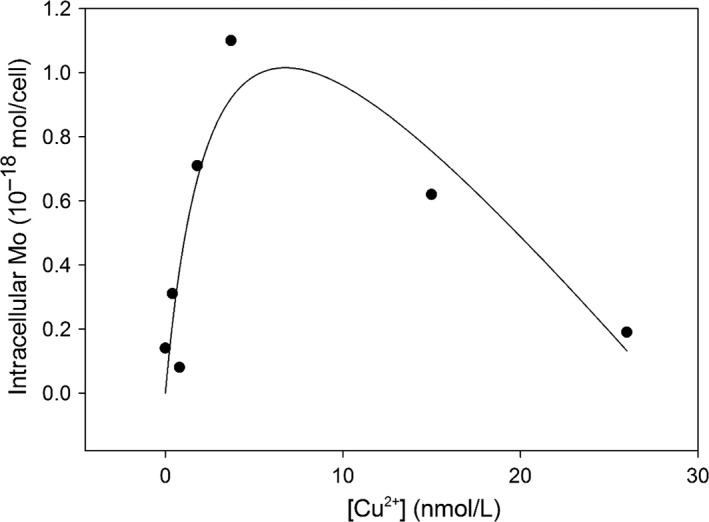
Kinetics of intracellular Mo in cultures exposed to [Cu^2+^]. The solid line represents the best fitting by a hyperbolic uptake minus linear expulsion

Both intracellular P and Mo of *P. tricornutum* accounted for >95% of the total (sum of surface‐adsorbed and internalized elemental fractions) in all cultures. Such results indicate that both anions are less adsorbed onto cell surfaces, and more subject to absorbed into cells. We observed that cellular P and Mo mainly exist as though in an intracellular compartment of *P. tricornutum*, which could be only correct in our case probably due to its specific cultural settings here. We would therefore discuss the intracellular P and Mo in this study. Our results show that elemental composition (intracellular Mo and P) was sensitive to Cu stress as indicated with large variability under different ambient Cu concentrations (Figures 5 and 6). Particularly, cellular P tends to decrease, while cellular Mo tends to increase under high levels of ambient Cu.

Both P and Mo are essential in cells for maintaining necessary physiological activities and growth. P is one of backbone elements involving the synthesis of basic structure such as membranes, while Mo is involved in forming active centers of enzymes catalyzing nitrate reduction and nitrogen fixation. In phytoplankton, trace elements commonly interact with each other during biological uptake due to ion competition and/or detoxification (Interlandi, 2002; Riedel & Sanders, 2003; Serra, Guasch, Admiraal, Van der Geest, & Van Beusekom, 2010; Wang & Dei, 2001). Hall, Healey, and Robinson (1989) observe that P is involved in excluding/eliminating toxic metals from cells in cultures of *Chlorella vulgaris*. Verma, Singh, and Singh (1993) show that the toxicity of Cu occurs in cyanobacteria once Cu induces phosphate starvation in the cells. Nalewajko and Olaveson (1994) suggest that Cu may directly reduce the phosphate uptake via impacting the permeability of the cell membranes. However, we observed with relatively high levels of cellular P in *P. tricornutum* during our experiments (Table 1), suggesting of little negative effects on the alga from ambient Cu. The levels of cellular P ranged between 50 and 120 × 10^−15^ mol/cell in all cultures (Figure 5). These cellular P levels were relatively higher than those total P values reported previously: 2 × 10^−15^ mol P/cell in *P. tricornutum* after 6 days in f‐media culture (Kuenzler & Ketchum, 1962), 4 × 10^−15^ mol P/cell in *Thalassiosira pseudonana* (Núñez‐Milland, Baines, Vogt, & Twining, 2010), and 10^−16^–10^−15^ mol P/cell in marine algae (Ho et al., 2003). The high levels of cellular P might be attributed to the efficient uptake mechanism of *P. tricornutum* under suitable culture settings. Such results suggest that excessive P ensured that enough P was absorbed into the cells for all metabolic requirements and even further storage of polyphosphates. The P‐replete conditions also indicate that the alga does not have requirements of taking up P ions by consuming extra energy from ambient waters.

Consistently, we observed that intracellular P slightly decreased with increasing [Cu^2+^], from 120 × 10^−15^ mol/cell initially to as low as 50 × 10^−15^ mol/cell in cultures exposed to Cu of 15–26 nmol/L under P‐replete conditions (Figure 4). A decrease of intracellular P by 50–60% suggested that the uptake of phosphate might be partly hindered in cultures exposed to increased [Cu^2+^] directly or indirectly. Peterson et al. (1984) reported similar results in cultures of green alga *Scendedesmus quadricauda*. Alternatively, the reduced levels of intracellular P might also reflect a net cellular P consumption in ambient P‐replete solutions.

The intracellular Mo ranged from 0.1 to 10 × 10^−18^ mol/cell in our cultures (Figure 6), which was similar to that in certain marine phytoplankton, for example, an average of ~3.3 × 10^−18^ mol/cell in 15 phytoplankton species (Ho et al., 2003), and 2–4 × 10^−18^ mol/cell in *Trichodesmium* (Tuit, Waterbury, & Ravizza, 2004), but slightly higher than that in *Crocosphaera* spp. (0.01–0.1 × 10^−18^ mol/cell, Tuit et al., 2004). In our culture, intracellular Mo was generally elevated in cultures exposed to moderate levels of Cu, and there was an initial increase followed by a gradual decrease of intracellular Mo (Figure 6). Such results reflect that *P. tricornutum* might be subject to a net uptake of Mo from ambient solutions. Basically, intracellular Mo increased sharply with increasing [Cu^2+^] until a peak of intracellular Mo (1.0 × 10^−18^ mol/cell) was reached in the culture exposed to [Cu^2+^] of 3.7 nmol/L, and then intracellular Mo decreased with further increasing [Cu^2+^] gradually, until 0.2 × 10^−18 ^mol/cell was reached in the culture exposed to [Cu^2+^] of 26 nmol/L. The general enhancement of cellular Mo in the cultures exposed to higher Cu levels suggested an increased uptake of Mo from the culture media, reflecting an increased requirement of Mo in the cells. A high requirement of the element is necessary to perform the counteracting activities ameliorating potential Cu toxicity in cells. There may be also some other specific roles of cellular Mo accumulation which needs to be further investigated. The dominance of intracellular Mo in total cellular Mo (>95%) suggested that the cell surface membrane was composed mainly of active binding sites transporting ${\text{MoO}}_{4}^{2-}$ into the cells, probably via ABC transporters (e.g., Wang, 2012). Once inside the cells, Mo may be actively involved in forming Cu–Mo or Cu–Mo–S organic complexes and neutralizing Cu toxicity (e.g., Suttle, 1987; Suttle & Field, 1983). The reason for a later decrease of intracellular Mo is unclear, and here, we simply attribute to the expulsion of specific Mo compounds as an impediment to Cu replacement by membrane transporters.

The uptake kinetics of Mo is modeled by combining a Michaelis–Menten hyperbolic adsorption with maximum saturation (Segel, 1976) and a linear cellular expulsion. The equation of Mo uptake into cells is as follows:

$$M=M_{\max}\times \left[{\text{Cu}}^{2+}\right]/\left(K_{m}+\left[{\text{Cu}}^{2+}\right]\right)-K_{e}\times \left[{\text{Cu}}^{2+}\right]$$

where M is the intracellular Mo concentration, *M*_max_ is the maximum saturation of intracellular Cu, *K*_m_ is the half‐saturation concentration, and *K*_e_ is the expulsion coefficient. Therefore, we obtained the values for *M*max, *K*_m_, and *K*_e_ by best least square fitting, as 2.18 (10^−18^ mol/cell), 3.13 (nmol/L), and 0.07 (10^−9^ L/cell) (*R* ^2^ = .79, Figure 6).

Phytoplankton species are capable of implementing several mechanisms to facilitate its growth. For example, *P. tricornutum* synthesizes organic molecules such as peptides and sulfhydryl binding extra metal ions (Gekeler, Grill, Winnacker, & Zenk, 1988; Morelli & Pratesi, 1997). Our study also showed that Cu accumulated mainly onto cell surfaces instead of directly being internalized. In addition, some phytoplankton/bacteria excrete organic substances complexing Cu extracellularly (Croot et al., 2000; Gordon et al., 1994), and other phytoplankton may also maintain a neutral pH in the cells, together with membrane of low permeability and of positive zeta, and membrane potentials repulsing extra metals (Rai, Rai, & Mallick, 1996). Our study suggested the possible involvement of intracellular elements (P and Mo) in these mechanisms. Relatively high levels of intracellular P ensure the production of polyphosphate bodies, complexing Cu ions, as suggested by Jensen et al. (1982) and Twiss and Nalewajko (1992). The active uptake of other elements such as Mo observed in our cultures exposed to [Cu^2+^] suggested the requirement of Mo in neutralizing Cu in cells. Mo is essential as involved in forming active centers of enzyme cofactors in phytoplankton (Wang, 2012). Increased uptake of Mo clearly facilitates algal metabolism and growth, and more importantly, Mo may directly react with Cu, to form metal‐binding organic molecules (e.g., Cu–Mo–S) inside cells, preventing the subsequent biding of plasma proteins from binding to Cu ions, thereby facilitating the excretion of Cu ions (Nickel, 2003).

### 3.4 Possible ecological consequences of Cu stress on the oceanic ecosystems

Our study basically showed three major impacts of C stress on phytoplankton: (1) Cu accumulation in cells; (2) alteration of elemental composition; (3) increased phytoplankton growth.

First, we also observed the increased cellular accumulation of Cu by *P. tricornutum* in our cultures, which suggests a potential for this diatom as a bioaccumulater to remove environmental Cu, as consistent with previous reports (Özkoc & Taylan, 2010). Our study further demonstrated that Cu uptake by *P. tricornutum* ranged from 25 to 50 × 10^−18^ mol/cell/72 hr and could reach as high as 50 × 10^−18^ mol/cell/72 hr in cultures exposed to Cu. Assuming that the cell abundance is 10^5^ cells/ml and the uptake rate of Cu is 25 × 10^−18^ mol/cell/72 hr in a bloom of *P. tricornutum*, we could then estimate that the bloom might cause a decrease of 2.5 nmol in 1 L of seawater within 72 hr. It should be noted that the actual uptake (cupric ions binding with cell membrane sites) was relatively fast, but the diffusion of ions from ambient waters to membrane sites was slow, and these processes were ignored in our calculation. Nevertheless, our calculation further showed the potential of *P. tricornutum* being used as an adsorbent or accumulator of Cu. High Cu accumulation in cells also remains toxic to the next trophic level in coastal ecosystems, as reported by Angel et al. (2015).

Second, we observed an alteration of elemental composition under Cu stress. The marine plankton has a relatively constrained elemental ratio of 106C:16N:1P atoms (Fleming, 1940; Redfield, 1934, 1958), which has been extensively used in marine biogeochemistry. The Redfield ratio concept has been extended to include micronutrients as well as trace metals (Bruland et al., 1991; Ho et al., 2003). On the other hand, the elemental stoichiometry of marine organisms may exhibit large fluxuations dependent upon physiological, ecological, biogeochemical, and evolutionary factors (Falkowski, 2000; Quigg, Irwin, & Finkel, 2011; Quigg et al., 2003; Twining & Baines, 2013). Elemental composition in the marine phytoplankton is also quite responsive to environmental parameters such as irradiance and nutrients (Finkel et al., 2006; Ho et al., 2003). In addition, the elemental composition of phytoplankton cells display a spatial difference in response to open ocean or coastal environments (Ho et al., 2003; Twining & Baines, 2013).

Our study further demonstrated that environmental changes such as Cu elevation could affect elemental composition (Mo/P) (Table 2). Indeed, our study demonstrated that the cellular ratio of Mo/P in *P. tricornutum* could be significantly elevated by as high as 10 times in cultures exposed to [Cu^2+^], with a range of 0.001–0.013 (Table 3). The results also indicated the use of elemental composition including Mo/P in any oceanic budget, and inferring ecological significances should be cautious as the ratios are generally sensitive and responsive to environmental changes.

**Table 2:** Comparison of cellular Mo/P ratios (mmol/mol) of marine phytoplankton in our study with previous research

Phytoplankton	Average Mo/P ratio (range)	References
5 phytoplankton species	0.075 (0.005–0.598)	Finkel et al. (2006)
20 phytoplankton species	0.1 (0.01–0.6)	Quigg et al. (2011)
15 phytoplankton species	0.033(0.01–0.12)	Ho et al. (2003)
*Phaeodactylum tricornutum*, <1 nmol/L Cu	0.001	Our study
0.25 μmol/L Cu	0.003	Our study
0.5 μmol/L Cu	0.002	Our study
1 μmol/L Cu	0.013	Our study
2 μmol/L Cu	0.013	Our study
8 μmol/L Cu	0.013	Our study
16 μmol/L Cu	0.004	Our study

**Table 3:** Cellular Cu in different phytoplankton species

Species	Cellular Cu (10^−18^ mol/cell)	References
*Synechococcus sp*.	0.2	Hudson and Morel (1993)
*Pyramimonas parkeae*	11	Quigg et al. (2003)
*Tetraselmis levis*	18	Hudson and Morel (1993)
*Amphidinium carterae*	2.9	Quigg et al. (2003)
*Emiliania huxleyi*	0.92	Quigg et al. (2003)
*Thalassiosira pseudonana*	3.8	Sunda and Huntsman (1995)
*Thalassiosira weissflogii*	21	Quigg et al. (2003)
*Phaeodactylum tricornutum*	1.3–12.8	Our study as in intracellular Cu

Finally, we observed with increased phytoplankton growth under Cu stress. The available literature suggests that modestly elevated concentrations of Cu can significantly affect the growth and primary production of coastal diatoms (Biswas, Bandyopadhyay, & Waite, 2013; Peers, Quesnel, & Price, 2005). Semeniuk et al. (2009) reported that high levels of dissolved Cu are responsible for the high abundance of phytoplankton in the subarctic northeast Pacific Ocean. Peers et al. (2005) observe comparable results in the Bering Sea, where an addition of 2 nmol/L Cu doubles the net growth rate of phytoplankton relative to those without Cu addition. Our study demonstrated that moderately increased ambient Cu (within the range used in our experiments: 0.25–16 μmol/L) can potentially enhance the growth of algae such as *P. tricornutum* (by as high as 40%). As a consequence, a bloom of phytoplankton in coastal waters there could be expected once the ambient toxic metals such as Cu are subject to increase chemically.

## Conflict of Interest

None declared.

## References

[ece32890-bib-0001] Ahner, B. A. , & Morel, F. M. M. (1995). Phytochelatin production in marine algae: II. Induction by various metals. Limnology and Oceanography, 40, 658–665.

[ece32890-bib-0002] Angel, B. M. , Simpson, S. L. , Chariton, A. A. , Stauber, J. L. , & Jolley, D. F. (2015). Time‐averaged copper concentrations from continuous exposures predicts pulsed exposure toxicity to the marine diatom, *Phaeodactylum tricornutum*: Importance of uptake and elimination. Aquatic Toxicology, 164, 1–9. 25911575 10.1016/j.aquatox.2015.04.008

[ece32890-bib-0003] Arredondo, M. , Martínez, R. , Núñez, M. T. , Ruz, M. , & Olivares, M. (2006). Inhibition of iron and copper uptake by iron, copper and zinc. Biological Research, 39, 95–102. 16629169 10.4067/s0716-97602006000100011

[ece32890-bib-0004] Baker, N. R. (2008). Chlorophyll fluorescence: A probe of photosynthesis in vivo. Annual Review of Plant Biology, 59, 89–113. 10.1146/annurev.arplant.59.032607.092759 18444897

[ece32890-bib-0005] Barón, M. , Arellano, J. B. , & Gorgé, J. L. (1995). Copper and photosystem II: A controversial relationship. Physiologia Plantarum, 94(1), 174–180.

[ece32890-bib-0006] Beck, A. J. , & Sañudo‐Wilhelmy, S. A. (2007). Impact of water temperature and dissolved oxygen on copper cycling in an urban estuary. Environmental Science and Technology, 41, 6103–6108. 17937288 10.1021/es062719y

[ece32890-bib-0007] Bentley‐Mowat, J. A. , & Reid, S. M. (1977). Survival of marine phytoplankton in high concentrations of heavy metals, and uptake of copper. Journal of Experimental Marine Biology and Ecology, 26(3), 249–264.

[ece32890-bib-0008] Biswas, H. , Bandyopadhyay, D. , & Waite, A. (2013). Copper addition helps alleviate iron stress in a coastal diatom: Response of *Chaetoceros gracilis* from the Bay of Bengal to experimental Cu and Fe addition. Marine Chemistry, 157, 224–232.

[ece32890-bib-0009] Bruland, K. W. , Donat, J. R. , & Hutchins, D. A. (1991). Interactive influences of bioactive trace metals on biological production in oceanic waters. Limnology and Oceanography, 36(8), 1555–1577.

[ece32890-bib-0010] Cid, A. , Herrero, C. , & Torres, E. (1995). Copper toxicity on the marine microalga *Phaeodactylum tricornutum*: Effects on photosynthesis and related parameters. Aquatic Toxicology, 31, 165–174.

[ece32890-bib-0011] Croot, P. L. , Moffett, J. W. , & Brand, L. E. (2000). Production of extracellular Cu complexing ligands by eucaryotic phytoplankton in response to Cu stress. Limnology and Oceanography, 45, 619–627.

[ece32890-bib-0012] Debelius, B. , Forja, J. M. , DelValls, Á. , & Lubián, L. M. (2009). Toxicity and bioaccumulation of copper and lead in five marine microalgae. Ecotoxicology and Environmental Safety, 72, 1503–1513. 19427695 10.1016/j.ecoenv.2009.04.006

[ece32890-bib-0013] Duerr, E. O. , Molnar, A. , & Sato, V. (1998). Cultured microalgae as aquaculture feeds. Journal of Marine Biotechnology, 6, 65–70.

[ece32890-bib-0014] Falkowski, P. G. (2000). Rationalizing elemental ratios in unicellular algae. Journal of Phycology, 36, 3–6.

[ece32890-bib-0016] Finkel, Z. K. , Quigg, A. , Raven, J. A. , Reinfelder, J. R. , Schofield, O. E. , & Falkowski, P. G. (2006). Irradiance and the elemental stoichiometry of marine phytoplankton. Limnology and Oceanography, 51(6), 2690–2701.

[ece32890-bib-0017] Fisher, N. S. , & Frood, D. (1980). Heavy metals and marine diatoms: Influence of dissolved organic compounds on toxicity and selection for metal tolerance among four species. Marine Biology, 59(2), 85–93.

[ece32890-bib-0018] Fleming, R. H. (1940). Composition of plankton and units for reporting populations and production (pp. 535–539). *Proceedings of the sixth pacific science congress of the pacific science association*. University of California Press.

[ece32890-bib-0019] Gao, K. , Xu, J. , Gao, G. , Li, Y. , Hutchins, D. A. , Huang, B. , … Riebesell, U. (2012). Rising CO_2_ and increased light exposure synergistically reduce marine primary productivity. Nature Climate Change, 2, 519–523.

[ece32890-bib-0020] Gekeler, W. , Grill, E. , Winnacker, E. L. , & Zenk, M. H. (1988). Algae sequester heavy metals via synthesis of phytochelatin complexes. Archives of Microbiology, 150(2), 197–202.

[ece32890-bib-0021] Gledhill, M. , Nimmo, M. , Hill, S. J. , & Brown, M. T. (1997). The toxicity of copper (II) species to marine algae, with particular reference to macroalgae. Journal of Phycology, 33(1), 2–11.

[ece32890-bib-0300] Gnassia‐Barelli, M. , Roméo, M. , Laumond, F. , & Pesando, D. (1978). Experimental studies on the relationship between natural copper complexes and their toxicity to phytoplankton. Marine Biology, 47, 15–19.

[ece32890-bib-0022] Gordon, A. S. , Howell, L. D. , & Harwood, V. (1994). Responses of diverse heterotrophic bacteria to elevated copper concentrations. Canadian Journal of Microbiology, 40(5), 408–411. 8069784 10.1139/m94-067

[ece32890-bib-0023] Grill, E. , Winnacker, E. L. , & Zenk, M. H. (1985). Phytochelatins: The principal heavy‐metal complexing peptides of higher plants. Science, 230, 674–676. 17797291 10.1126/science.230.4726.674

[ece32890-bib-0024] Hall, A. (1981). Copper accumulation in copper‐tolerant and non‐tolerant populations of the marine fouling alga, *Ectocarpus siliculosus* (Dillw.) Lyngbye. Botanica Marina, 24, 223–228.

[ece32890-bib-0025] Hall, J. , Healey, F. P. , & Robinson, G. G. C. (1989). The interaction of chronic copper toxicity with nutrient limitation in two chlorophytes in batch culture. Aquatic Toxicology, 14, 1–14.

[ece32890-bib-0026] Ho, T. Y. , Quigg, A. , Finkel, Z. V. , Milligan, A. J. , Wyman, K. , Falkowski, P. G. , & Morel, F. M. M. (2003). The elemental composition of some marine phytoplankton. Journal of Phycology, 39, 1145–1159.

[ece32890-bib-0027] Hoffmann, L. , Breitbarth, E. , Boyd, P. W. , & Hunter, K. A. (2012). Influence of ocean warming and acidification on trace metal biogeochemistry. Marine Ecology Progress Series, 470, 191–205.

[ece32890-bib-0028] Hudson, R. J. , & Morel, F. M. M. (1990). Iron transport in marine phytoplankton: Kinetics of cellular and medium coordination reactions. Limnology and Oceanography, 35(5), 1002–1020.

[ece32890-bib-0029] Hudson, R. J. , & Morel, F. M. M. (1993). Trace metal transport by marine microorganisms: Implications of metal coordination kinetics. Deep Sea Research Part I, 40, 129–150.

[ece32890-bib-0030] Interlandi, S. J. (2002). Nutrient‐toxicant interactions in natural and constructed phytoplankton communities: Results of experiments in semicontinuous and batch culture. Aquatic Toxicology, 61, 35–51. 12297369 10.1016/s0166-445x(02)00016-4

[ece32890-bib-0031] Janik, E. , Maksymiec, W. , & Gruszecki, W. I. (2010). The photoprotective mechanisms in *Secale cereale* leaves under Cu and high light stress condition. Journal of Photochemistry and Photobiology B: Biology, 101(1), 47–52. 10.1016/j.jphotobiol.2010.06.010 20655756

[ece32890-bib-0032] Jegerschöld, C. , Arellano, J. B. , Schröder, W. P. , Van Kan, P. J. M. , Barón, M. , & Styring, S. (1995). Copper (II) inhibition of electron transport through photosystem II studied by EPR spectroscopy. Biochemistry, 34, 12747–12754. 7548028 10.1021/bi00039a034

[ece32890-bib-0033] Jensen, T. E. , Baxter, M. , Rachlin, J. W. , & Jani, V. (1982). Uptake of heavy metals by *Plectonema boryanum* (cyanophyceae) into cellular components, especially polyphosphate bodies: An X‐ray energy dispersive study. Environmental Pollution Series A, Ecological and Biological, 27, 119–127.

[ece32890-bib-0034] Knauer, K. , Behra, R. , & Sigg, L. (1997). Adsorption and uptake of copper by the green alga *Scenedesmus subspicatus* (Chlorophyta). Journal of Phycology, 33, 596–601.

[ece32890-bib-0035] Kozelka, P. B. , & Bruland, K. (1998). Chemical speciation of dissolved Cu, Zn, Cd, Pb in Narragansett Bay, Rhode Island. Marine Chemistry, 60, 267–282.

[ece32890-bib-0036] Kuenzler, E. J. , & Ketchum, B. H. (1962). Rate of phosphorus uptake by *Phaeodactylum tricornutum* . The Biological Bulletin, 123(1), 134–145.

[ece32890-bib-0037] Leao, P. N. , Vasconcelos, M. T. S. D. , & Vasconcelos, V. M. (2007). Role of marine cyanobacteria in trace metal bioavailability in seawater. Microbial Ecology, 53, 104–109. 17186147 10.1007/s00248-006-9153-6

[ece32890-bib-0038] Les, A. , & Walker, R. W. (1984). Toxicity and binding of copper, zinc, and cadmium by the blue‐green alga, *Chroococcus paris* . Water, Air, and Soil Pollution, 23(2), 129–139.

[ece32890-bib-0039] Levy, J. L. , Angel, B. M. , Stauber, J. L. , Poon, W. L. , Simpson, S. L. , Cheng, S. H. , & Jolley, D. F. (2008). Uptake and internalisation of copper by three marine microalgae: Comparison of copper‐sensitive and copper‐tolerant species. Aquatic Toxicology, 89, 82–93. 18639348 10.1016/j.aquatox.2008.06.003

[ece32890-bib-0040] Levy, J. L. , Stauber, J. L. , & Jolley, D. F. (2007). Sensitivity of marine microalgae to copper: The effect of biotic factors on copper adsorption and toxicity. Science of the Total Environment, 387(1), 141–154. 17765293 10.1016/j.scitotenv.2007.07.016

[ece32890-bib-0041] Li, Y. , Gao, K. , Villafañe, V. E. , & Helbling, E. W. (2012). Ocean acidification mediates photosynthetic response to UV radiation and temperature increase in the diatom *Phaeodactylum tricornutum* . Biogeosciences, 9, 7197–7226.

[ece32890-bib-0042] Li, X. , Wai, O. W. H. , Li, Y. S. , Coles, B. J. , & Ramsey, M. H. (2000). Heavy metal distribution in sediment profiles of the Pearl River estuary, South China. Applied Geochemistry, 15(5), 567–581.

[ece32890-bib-0043] Li, Y. H. , Xu, J. T. , & Gao, K. S. (2014). Light‐modulated responses of growth and photosynthetic performance to ocean acidification in the model diatom *Phaeodactylum tricornutum* . PLoS ONE, 9(5), e96173. 24828454 10.1371/journal.pone.0096173 PMC4020747

[ece32890-bib-0044] Lidon, F. C. , & Henriques, F. S. (1993). Effects of copper toxicity on growth and the uptake and translocation of metals in rice plants. Journal of Plant Nutrition, 16, 1449–1464.

[ece32890-bib-0045] Mehta, S. K. , & Gaur, J. P. (1999). Heavy‐metal‐induced proline accumulation and its role in ameliorating metal toxicity in *Chlorella vulgaris* . New Phytologist, 143, 253–259.

[ece32890-bib-0046] Millero, F. J. (2009). Effect of ocean acidification on the speciation of metals in seawater. Oceanography, 22(4), 72–85.

[ece32890-bib-0048] Morel, F. M. , & Price, N. M. (2003). The biogeochemical cycles of trace metals in the oceans. Science, 300, 944–947. 12738853 10.1126/science.1083545

[ece32890-bib-0049] Morelli, E. , & Pratesi, E. (1997). Production of phytochelatins in the marine diatom *Phaeodactylum tricornutum* in response to copper and cadmium exposure. Bulletin of Environmental Contamination and Toxicology, 59(4), 657–664. 9307434 10.1007/s001289900530

[ece32890-bib-0050] Nalewajko, C. , & Olaveson, M. M. (1994). Differential responses of growth, photosynthesis, respiration, and phosphate uptake to copper in copper‐tolerant and copper‐intolerant strains of *Scenedesmus acutus* (Chlorophyceae). Canadian Journal of Botany, 73, 1295–1303.

[ece32890-bib-0051] Nickel, W. (2003). The mystery of nonclassical protein secretion, a current view on cargo proteins and potential export routes. European Journal of Biochemistry, 270(10), 2109–2119. 12752430 10.1046/j.1432-1033.2003.03577.x

[ece32890-bib-0052] Nielsen, H. D. , Brownlee, C. , Coelho, S. M. , & Brown, M. T. (2003). Inter‐population differences in inherited copper tolerance involve photosynthetic adaptation and exclusion mechanisms in *Fucus serratus* . New Phytologist, 160(1), 157–165. 10.1046/j.1469-8137.2003.00864.x 33873539

[ece32890-bib-0053] Núñez‐Milland, D. R. , Baines, S. B. , Vogt, S. , & Twining, B. S. (2010). Quantification of phosphorus in single cells using synchrotron X‐ray fluorescence. Journal of Synchrotron Radiat., 17(4), 560–566. 10.1107/S0909049510014020 PMC3025539 20567089

[ece32890-bib-0054] Özkoc, H. B. , & Taylan, Z. S. (2010). Assessment of various parameters of metal biology in marine microalgae *Phaeodactylum Tricornutum* and *Dunaliella Tertiolecta* . Fresenius Environmental Bulletin, 19(12), 2981–2986.

[ece32890-bib-0055] Peers, G. , Quesnel, S. A. , & Price, N. M. (2005). Copper requirements for iron acquisition and growth of coastal and oceanic diatoms. Limnology and Oceanography, 50(4), 1149–1158.

[ece32890-bib-0056] Peterson, H. G. , Healey, F. P. , & Wagemann, R. (1984). Metal toxicity to algae: A highly pH dependent phenomenon. Canadian Journal of Fisheries and Aquatic Sciences, 41(6), 974–979.

[ece32890-bib-0057] Pistocchi, R. , Mormile, M. A. , Guerrini, F. , Isani, G. , & Boni, L. (2000). Increased production of extra‐and intracellular metal‐ligands in phytoplankton exposed to copper and cadmium. Journal of Applied Phycology, 12(3–5), 469–477.

[ece32890-bib-0058] Quigg, A. , Finkel, Z. V. , Irwin, A. J. , Rosenthal, Y. , Ho, T. Y. , Reinfelder, J. R. , … Falkowski, P. G. (2003). The evolutionary inheritance of elemental stoichiometry in marine phytoplankton. Nature, 425, 291–294. 13679916 10.1038/nature01953

[ece32890-bib-0059] Quigg, A. , Irwin, A. J. , & Finkel, Z. V. (2011). Evolutionary inheritance of elemental stoichiometry in phytoplankton. Proceedings of the Royal Society B, 278, 526–534. 20826483 10.1098/rspb.2010.1356 PMC3025679

[ece32890-bib-0060] Rai, L. C. , Rai, P. K. , & Mallick, N. (1996). Regulation of heavy metal toxicity in acid‐tolerant *Chlorelia*: Physiological and biochemical approaches. Environmental and Experimental Botany, 36(1), 99–109.

[ece32890-bib-0061] Raskin, I. , Kumar, P. B. A. N. , Dushenkov, S. , & Salt, D. E. (1994). Bioconcentration of heavy metals by plants. Current Opinion in Biotechnology, 5, 285–290.

[ece32890-bib-0062] Redfield, A. C. (1934).On the proportions of organic derivatives in sea water and their relation to the composition of plankto In: Daniel R. J. (Ed.), James Johnstone Memorial Volume (pp. 176–192), Liverpool: Liverpool Univ. Press.

[ece32890-bib-0063] Redfield, A. C. (1958). The biological control of the chemical factors in the environment. American Scientist, 46, 205–221.

[ece32890-bib-0064] Reed, R. H. , & Gadd, G. M. (1990). Metal tolerance in eukaryotic and prokaryotic algae In: Shaw A. J. (Ed.), Heavy metal tolerance in plants: Evolutionary aspects (pp. 105–118). Boca Raton, FL: CRC Press.

[ece32890-bib-0065] Riedel, G. F. , & Sanders, J. G. (2003). The interrelationships among trace element cycling, nutrient loading, and system complexity in estuaries: A mesocosm study. Estuaries, 26, 339–351.

[ece32890-bib-0066] Rijstenbil, J. W. , & Wijnholds, J. A. (1996). HPLC analysis of nonprotein thiols in planktonic diatoms: Pool size, redox state and response to copper and cadmium exposure. Marine Biology, 127, 45–54.

[ece32890-bib-0067] Schröder, W. P. , Arellano, J. B. , Bitter, T. , Barón, M. , Eckert, H. J. , & Regner, G. (1994). Flash‐induced absorption spectroscopy studies of copper interaction with photosystem II in higher plants. Journal of Biological Chemistry, 269, 32865–32870. 7806512

[ece32890-bib-0068] Segel, I. H. (1976). Biochemical calculations (2nd ed.). London: John Wiley & Sons.

[ece32890-bib-0069] Semeniuk, D. M. , Cullen, J. T. , Johnson, W. K. , Gagnon, K. , Ruth, T. J. , & Maldonado, M. T. (2009). Plankton copper requirements and uptake in the subarctic Northeast Pacific Ocean. Deep Sea Research Part I, 56(7), 1130–1142.

[ece32890-bib-0070] Serra, A. , Guasch, H. , Admiraal, W. , Van der Geest, H. G. , & Van Beusekom, S. A. M. (2010). Influence of phosphorus on copper sensitivity of fluvial periphyton: The role of chemical, physiological and community‐related factors. Ecotoxicology, 19, 770–780. 20024616 10.1007/s10646-009-0454-7 PMC2844973

[ece32890-bib-0071] Sunda, W. G. (1975). The relationship between cupric ion activity and the toxicity of copper to phytoplankton. Ph.D. thesis. MIT/WHOI.

[ece32890-bib-0072] Sunda, W. G. (2012). Feedback interactions between trace metal nutrients and phytoplankton in the ocean. Frontiers in Microbiology, 3, 204. doi: 10.3389/fmicb.2012.00204 22701115 10.3389/fmicb.2012.00204 PMC3369199

[ece32890-bib-0073] Sunda, W. G. , & Huntsman, S. A. (1988). Interactions among Cu^2+^, Zn^2+^, and Mn^2+^ in controlling cellular Mn, Zn, and growth rate in the coastal alga *Chlamydomonas* . Limnology and Oceanography, 43(6), 1055–1064.

[ece32890-bib-0074] Sunda, W. G. , & Huntsman, S. A. (1995). Regulation of copper concentration in the oceanic nutricline by phytoplankton uptake and regeneration cycles. Limnology and Oceanography, 40, 132–137.

[ece32890-bib-0075] Sunda, W. G. , & Lewis, J. A. M. (1978). Effect of complexation by natural organic ligands on the toxicity of copper to a unicellular alga, *Monochrysis lutheri* . Limnology and Oceanography, 23(5), 870–876.

[ece32890-bib-0076] Suttle, N. F. (1987). The absorption, retention and function of minor nutrients. In: Proc. 2nd Int. Symp. the Nutrition of Herbivores (pp. 333–361). Brisbane, Sydney: Academic.

[ece32890-bib-0077] Suttle, N. F. , & Field, A. C. (1983). Effects of dietary supplements of thiomolybdates on copper and molybdenum metabolism in sheep. Journal of Comparative Pathology, 93, 379–389. 6886084 10.1016/0021-9975(83)90025-7

[ece32890-bib-0078] Tang, D. , & Morel, F. M. (2006). Distinguishing between cellular and Fe‐oxide‐associated trace elements in phytoplankton. Marine Chemistry, 98(1), 18–30.

[ece32890-bib-0080] Tovar‐Sanchez, A. , Sañudo‐Wilhelmy, S. A. , Garcia‐Vargas, M. , Weaver, R. S. , Popels, L. C. , & Hutchins, D. A. (2003). A trace metal clean reagent to remove surface‐bound iron from marine phytoplankton. Marine Chemistry, 82(1), 91–99.

[ece32890-bib-0081] Tuit, C. , Waterbury, J. , & Ravizza, G. (2004). Diel variation of molybdenum and iron in marine diazotrophic cyanobacteria. Limnology and Oceanography, 49(4), 978–990.

[ece32890-bib-0082] Twining, B. S. , & Baines, S. B. (2013). The trace metal composition of marine phytoplankton. Annual Review of Marine Science, 5, 191–215. 10.1146/annurev-marine-121211-172322 22809181

[ece32890-bib-0400] Twiss, M. R. , & Nalewajko, C . (1992). Influence of phosphorus nutrition on copper toxicity to three strains of Scenedesmus acutus (Chlorophyceae) J Phycology, 28, 291–298.

[ece32890-bib-0083] Vasconcelos, M. T. S. D. , & Leal, M. F. C. (2008). Exudates of different marine algae promote growth and mediate trace metal binding in *Phaeodactylum tricornutum* . Marine Environment Research, 66(5), 499–507. 10.1016/j.marenvres.2008.07.002 18829098

[ece32890-bib-0084] Verma, S. K. , Singh, R. K. , & Singh, S. P. (1993). Copper toxicity and phosphate utilization in the cyanobacterium *Nostoc calcicola* . Bulletin of Environment Contamination and Toxicology, 50, 192–198. 10.1007/BF00191721 8422521

[ece32890-bib-0085] Wang, D. (2012). Redox chemistry of molybdenum in natural waters and its involvement in biological evolution. Frontiers in Microbiology, 3, 1–7. 23267355 10.3389/fmicb.2012.00427 PMC3528336

[ece32890-bib-0086] Wang, W. X. , & Dei, R. C. (2001). Metal uptake in a coastal diatom influenced by major nutrients (N, P, and Si). Water Research, 35(1), 315–321. 11257887 10.1016/s0043-1354(00)00256-6

[ece32890-bib-0087] Wang, D. , Lin, W. , Yang, X. , Zhai, W. , Dai, M. , & Chen, C. T. A. (2012). Occurrences of dissolved trace metals (Cu, Cd, and Mn) in the Pearl River Estuary (China), a large river‐groundwater‐estuary system. Continental Shelf Research, 50(51), 54–63.

[ece32890-bib-0089] Whitfield, M. (2001). Interactions between phytoplankton and trace metals in the ocean. Advances in Marine Biology, 41, 1–128.

[ece32890-bib-0090] Wikfors, G. H. , & Ukeles, R. (1982). Growth and adaptation of estuarine unicellular algae in media with excess copper, cadmium or zinc, and effects of metal‐contaminated algal food on *Crassostrea virginica* larvae. Marine Ecology Progress Series, 7(2), 91–106.

[ece32890-bib-0092] Yruela, A. J. , Pueyo, J. J. , Alonso, P. J. , & Picorel, R. (1996). Photoinhibition in photosystem II from higher plants. Journal of Biological Chemistry, 271, 27408–27415. 8910320 10.1074/jbc.271.44.27408

